# The Regenerative Potential of Female Skeletal Muscle upon Hypobaric Hypoxic Exposure

**DOI:** 10.3389/fphys.2016.00303

**Published:** 2016-07-14

**Authors:** Rosa Mancinelli, Ester S. Di Filippo, Vittore Verratti, Stefania Fulle, Luana Toniolo, Carlo Reggiani, Tiziana Pietrangelo

**Affiliations:** ^1^Department of Neuroscience, Imaging and Clinical Sciences, G. d'Annunzio University of Chieti-PescaraChieti, Italy; ^2^Laboratory of Functional Evaluation, University ‘G. d'Annunzio’, Chieti-PescaraChieti, Italy; ^3^Interuniversity Institute of MyologyChieti, Italy; ^4^Department of Anatomy and Physiology, University of PaduaPadua, Italy

**Keywords:** hypoxia, miRNA, oxidative stress, satellite cells, single fiber, women

## Abstract

**Aim:** The aim of this study was to determine whether a 14-day trekking expeditions, in high altitude hypoxic environment, triggers redox disturbance at the level of satellite cells (adult stem cells) in young women.

**Methods:** We collected muscle biopsies from *Vastus Lateralis* muscle for both single fiber analysis and satellite cells isolation. The samples collected before (PRE-Hypoxia) and after (POST-Hypoxia) the trekking in the Himalayas were compared. Satellite cells were investigated for oxidative stress (oxidant production, antioxidant enzyme activity, and lipid damage), mitochondrial potential variation, gene profile of *HIF*, and myogenic transcription factors (Pax7, MyoD, myogenin), and miRNA expression (miR-1, miR-133, miR-206).

**Results:** The nuclear domain analysis showed a significant fusion and consequent reduction of the Pax7^+^ satellite cells in the single mature fibers. The POST-Hypoxia myoblasts obtained by two out of six volunteers showed high superoxide anion production and lipid peroxidation along with impaired dismutase and catalase and mitochondrial potential. The transcription profile and miRNA expression were different for oxidized and non-oxidized cells.

**Conclusions:** The present study supports the phenomenon of hypobaric-hypoxia-induced oxidative stress and its role in the impairment of the regenerative capacity of satellite cells derived from the *V. Lateralis* muscle of young adult female subjects.

## Introduction

The hypobaric hypoxia created by environmental conditions as during high altitude exposure, provides a strenuous oxygen supply restriction that affects human skeletal muscle, and leads to its specific adaptation. The decreased oxygen supply become exacerbated when physical exercise is performed during high altitude exposure (Verratti et al., 2015).

It is known that chronic local hypoxia in skeletal muscle leads to a negative regulation of gene and protein balance and to a loss of muscle mass by means of hypoxia inducible factor (HIF) that initiates transcription of HIF-responsive genes (Chandel et al., 2000).

Concerning the muscle mass loss in hypoxia, there are other opposite evidence that sojourn at 5000 m along with moderate physical activity determined a switch of fiber phenotype from fast to slow in male and female lower limb with positive adaptation on muscle mass (Mancinelli et al., 2011; Tam et al., 2016). Other studies, suggested that a male muscle remodeling could depend on fusion of activated human myoblasts with the mature fibers (Doria et al., 2011; Mancinelli et al., 2011).

Indeed, the skeletal muscle tissue remodeling is due to the satellite cells, adult stem cells, that upon activation are able to fuse with existing fibers, or to form new ones. Moreover, once activated, these cells proliferate (myoblasts), and differentiate (myotubes) rebuilding the muscle tissue (Ceafalan et al., 2014; Verdijk, 2014; Verdijk et al., 2014). This process, named myogenesis, occurs both *in vivo* and *in vitro* conditions.

Different stimuli can both negatively or positively regulate the regenerative capacity of satellite cells, such as hypoxia, oxidative stress, and physical exercise (Di Carlo et al., 2004; Pietrangelo et al., 2015).

Mitochondria and oxygen are particularly involved in skeletal muscle adaptation to stimuli as hypobaric hypoxia and exercise (Hoppeler et al., 2003). Indeed, mitochondria, the cellular oxygen sensors, manage the reactive oxygen species production and their detoxification (Kietzmann et al., 2000).

Paradoxically, it has been proposed that hypobaric-hypoxia provokes alteration of mitochondrial activity that results an accumulation of reactive oxygen species (ROS) and oxidative stress (Vanden Hoek et al., 1998; Abele et al., 2002; Barbieri and Sestili, 2012; Barbieri et al., 2014). ROS production in hypobaric hypoxia is still a challenging process to be investigated (Archer and Michelakis, 2002) on satellite cell pool that can be at the same time the source and the target of oxidative stress. In both cases the result is an impaired muscle regeneration.

It has been demonstrated that oxidative stress impairs the ability of satellite cells to differentiate by damaging macromolecules as lipids, proteins, and nucleic acids (Fulle et al., 2005; Beccafico et al., 2007; Pietrangelo et al., 2009; Di Filippo et al., 2016).

Furthermore, recent literature suggests that micro-(mi)RNAs, the post-transcriptional RNAs, can be regulated by oxidative stress (Magenta et al., 2013). The muscle specific miRNAs regulate gene expression during skeletal muscle adaptation by influencing cell proliferation, apoptosis, and differentiation (Eisenberg et al., 2009; Huang et al., 2012; La Rovere et al., 2014).

Evidence of physiological adaptation to high altitude has largely been investigated on men despite female subjects even if a sex as well as an individual dependent-response variability to hypobaric hypoxia seems to exist (Chapman et al., 1998; Tam et al., 2016).

Considering all of these aspects, we asked whether the satellite cell pool of female subjects is affected by the oxidative imbalance that might be caused by hypobaric hypoxia, as during Gokio Kumbu/Amadablam 2012 expedition, and the involvement of miRNA regulation.

## Materials and methods

We enrolled in the Laboratory of Functional Evaluation seven healthy sedentary female subjects (#1–7) of childbearing age (mean age, 36.3 ± 7.1 years old, body weight 65.8 ± 11.7 kg, body mass index 24.3 ± 4.0 kg m^−2^) who were generally used to a sedentary life-style to serve as subjects to the study known as GOKYO KHUMBU/AMA DABLAM TREK 2012, carried out during the same expedition (details of the expedition are reported in Tam et al., 2016). The women 4 months previously were engaged in a specific trekking at low altitude in Abruzzo Mountains, to familiarize, and exercise them to trek at high altitude (Tam et al., 2016) and before this experience, we performed tiny percutaneous needle biopsy of *Vastus lateralis* muscle.

The subjects provided their written informed consent, and the study was conducted according to the Helsinki Declaration (as amended in 2000) and approved by the Ethics Committee of the “G. d'Annunzio” University of Chieti–Pescara, Italy (protocol no. 773 COET).

### Skeletal muscle needle biopsy

Tiny percutaneous needle biopsies from the *V. Lateralis* muscle were performed as described by Pietrangelo et al. (2011). The experimental time points were at the beginning of the study, before initiating any exercise training (PRE-Hypoxia), and 5 days after the return from the 14 days of moderate exercise training at high altitude (POST-Hypoxia) performed in the Himalayas during the Gokio Kumbu expedition. We obtained three tiny muscle samples each dedicated to single experimental set: one bioptic sample was dedicated to analysis of immunohistochemistry on single fiber, another bioptic sample for obtaining satellite cell populations as described in the current work. Of note, we obtained a third bioptic sample used to perform muscle fiber high-resolution respirometry data already published in Tam et al. (2016).

### Satellite cell populations and myogenicity

The human adult myogenic precursor cells, also named myoblasts, were obtained in cultures after their migration from muscle explants in which they resided as satellite cells. The myoblasts were expanded and differentiated in appropriate medium, as described previously (Fulle et al., 2005; Di Filippo et al., 2016). The percentages of myogenicity of cell cultures were determined using immunocytochemistry assays, with desmin as the marker (a cytoskeleton protein of the intermediate filaments of myogenic populations), and the LSAB+ System-AP Universal kits (Cat. No. K0678; DAKO, Dakocytomation, Glostrup, Denmark). The percentages of desmin-positive myoblasts were calculated as the ratio between the number of desmin-positive cells and the total number of cells. Differentiation was determined by counting the numbers of nuclei in myotubes after 7 days of differentiation, as percentages with respect to the total number of nuclei, with the ratio between these two values (nuclei in myotubes/ total nuclei × 100) giving the Fusion Index (Pietrangelo et al., 2009). We considered uniquely the myotubes positive to the primary antibody against myosin heavy chain (MyHC, MF20 monoclonal antibody; diluted 1:50; from Developmental Studies Hybridoma Bank, University of Iowa, Iowa City, IA, USA) that contained at least three nuclei.

### Immunohistochemistry on single fiber

Muscle sample from each subject, well-preserved considering the fiber ultrastructural properties (Pietrangelo et al., 2013), were manually dissected to obtain single fibers (about 100 fibers equally obtained by each biopsy). Unfortunately, the fibers obtained from subject #3 were not suitable.

The myonuclei on single fiber were counted to determine the nuclear domain. On 61 of these fibers were also counted Pax7^+^ nuclei by immune fluorescence technique. The fibers, lying in plate coated with Sylgard, blocked well-aligned, were fixed with 4% paraformaldehyde in PBS for 20 min at room temperature and then permeabilized with 0.1% Triton X-100 in PBS. To avoid unspecific antibody binding, the fibers were incubated in 10% normal goat serum for 30 min and then with Mouse anti Pax-7 (RandD System) monoclonal antibody in PBS (1:400) applied at 4°C overnight. After 3 washes with PBS (10 min each), the fluorescent secondary Alexa-568 anti-mouse antibody (Molecular Probes) was incubated for 2 h at room temperature. To localize the nuclei, single fibers were stained with Hoestch (25 μg/ml; SIGMA) for 10 min. The confocal microscope (VICO; Nikon) was used to acquire fluorescent images. The myonuclear density was determined as number of nuclei in constant fiber volume (10^6^ μm^3^). The nuclear domain was calculated by dividing fiber volume for the number of nuclei; the satellite cells with Pax7^+^ nuclei were expressed as percentage referred to 100 nuclei. The data were reported as mean ± standard error.

### Reactive oxygen species production

We used the assay based on the dye nitroblue tetrazolium chloride (NBT, Cat.No. N6639; Sigma-Aldrich) and its reduction by the ${\text{O}}_{2}^{•-}$ into formazan, using a spectrophotometer for the absorbance at 550 nm (Microplate 257 spectrometer; SPECTRAmax 190, Molecular Devices, Sunnyvale, CA, USA), such that the greater the ${\text{O}}_{2}^{•-}$ level, the greater the absorbance. The cells (1 × 10^6^) were detached, centrifuged at 170 × *g* for 5 min, resuspended in 1 mL NBT at 1 mg ml^−1^ in 0.9% aqueous NaCl, and incubated for 3 h at 37°C. Then, the cells were centrifuged at 100 × *g* for 10 min, resuspended in 1 ml DMSO, and left for 20 min at 37°C; finally, the NBT absorbance was determined. Unfortunately, the muscle biopsy obtained from subject #7 did not produce sufficient numbers of satellite cells for the superoxide anion (${\text{O}}_{2}^{•-}$) determination.

The analysis of the ROS was conducted using the dye 2,7-dichlorofluorescein diacetate (DCFH-DA, Cat. No. D6883; Sigma). The cells (1000 per well) were plated and grown in 96-well microplates, and incubated with 10 μM DCFH-DA for 30 min at 37°C in sterile normal external solution (140 mM NaCl, 2.8 mM KCl, 2 mM CaCl_2_, 2 mM MgCl_2_, 10 mM glucose, 10 mM Hepes, pH 7.3). The fluorescence of the dye accumulated in the cytoplasm (DCF) was determined at 530 nm (excitation, 490 nm) using a SPECTRAmax fluorometer (Gemini XS; Molecular Devices Toronto, ON, Canada). The analysis was conducted using the SOFTmax Pro software. We stimulated the cells with 100 nM H_2_O_2_ to evaluate their responses to an oxidant (Menghini et al., 2011).

### Antioxidant enzyme activity

The antioxidant enzymes analyzed were superoxide dismutase and catalase. The assays were performed using the cytosolic fraction.

### Superoxide dismutase

The activity of superoxide dismutase (SOD) is direct against ${\text{O}}_{2}^{•-}$. SOD catalyzes a disproportionation reaction where a first ${\text{O}}_{2}^{•-}$ is oxidized and the second molecule is reduced, turning two molecules of superoxide into O_2_ and H_2_O_2_. The enzymatic activity was determined according to Fulle et al. (2000). The final assay volume was 1 ml and contained 20 mM Na_2_CO_3_ buffer pH 10, 10 mM Cytochrome c, 1 mM Xanthine and Xanthine Oxidase. Xanthine-xanthine oxidase is the ${\text{O}}_{2}^{•-}$ generation system. As the xanthine oxidase activity varies, the amount used for the assay was such that produced a rate of cytochrome c reduction, at 550 nm, of 0.025 per min without SOD addiction. The assay was performed at 550 nm for 10 min. The SOD units were calculated considering that 1 SOD unit is defined as the quantity that inhibits the rate of cytochrome c reduction by 50%.

### Catalase

The reaction for which catalase (Cat) is best known is the “catalatic” reaction, in which H_2_O_2_ oxidizes the heme iron of the resting enzyme to form an oxyferryl group with a π-cationic porphyrin radical (Kirkman and Gaetani, 2007). This step is followed by oxidation of a second molecule of H_2_O_2_. Catalase forms two molecules of H_2_O and O_2_, starting from two molecules of H_2_O_2_. Catalase activity was determined, according to Greenwald (1985), by the decrease in absorbance due to H_2_O_2_ consumption (ε = −0.04 mM^−1^ cm^−1^) measured at 240 nm. The final reaction volume was 1 ml and contained 100 mM Na-phosphate buffer pH 7.0, 12 μM H_2_O_2_, and 70 μg of sample proteins. The reaction was followed for 1 min and the Cat activity was expressed in μmol/minute/mg proteins.

### Lipid peroxidation assay

Malondialdehyde (MDA) forms an adduct with thiobarbituric acid (TBA), which is measurable using a spectrophotometer. For lipid peroxidation analysis, we used the OXItek TBARS Assay Kit (ZeptoMetrix Corporation, Buffalo, NY). We mixed 100 μl of SDS and 100 μl of samples obtained from sonicated myoblasts (in PBS) and then added 2.5 ml of TBA Buffer Reagent. Samples were incubated at 95°C for 1 h. The reaction was stopped by cooling in an ice bath for 10 min. After centrifugation at 3000 rpm for 15 min, the supernatant absorbance was read at 532 nm. The amount of MDA was calculated using a standard curve. Results are expressed as nmol of MDA per mg of protein.

### Variations in the transmembrane mitochondrial potential

The mitochondrial membrane potential was determined using the JC-1 dye (5,5′,6, 6′-tetracloro-1,1′,3,3′-tetraethylbenzimidazolylcarbocianine iodide/ chloride; Molecular Probes), which is a cationic dye that accumulates in the mitochondria. When the mitochondrial potential is high, as in normal cells, JC-1 aggregates into dimers that emit red fluorescence (aggregated J: excitation/ emission, 560/595 nm). When the membrane potential is low, as in the presence of oxidative stress, JC-1 forms monomers that emit green fluorescence (excitation/emission, 488/522 nm), with decreased red fluorescence. The ratio of the red/green fluorescence depends exclusively on the mitochondrial potential, with no effects of other factors (such as dimension, volume, shape, mitochondrial density). The cells were plated into 96-well plates, incubated with 10 μg ml^−1^ JC-1 for 15 min at 37°C, and read using a Fluorometer SPECTRAmax equipped with SoftMax Pro (Gemini XS, Molecular Devices Toronto, ON, Canada) (Nuydens et al., 1999). The acquired fluorescence values are reported as means ± SEM of the red/ green fluorescence ratios of samples with respect to control: f(r/g)/f(r/g)_c_ (Morabito et al., 2010). The JC-1 dye ratio between the inner and outer mitochondrial membrane potentials relates to the mitochondrial depolarization after an oxidant insult, such as H_2_O_2_.

### Quantitative gene profile of myogenic transcription factors and expression of hypoxia inducible factor

We investigated undifferentiated and 7 day differentiated cells. Total RNA was purified using standard protocols with Trizol (#T9424; Sigma-Aldrich, Milan, Italy). After spectrophotometric and electrophoretic quantification and analysis, 1 μg pure RNA was processed to obtain cDNA (High Capacity cDNA Reverse Transcription, #4368814; Applied Biosystems), and 100 ng cDNA was used for the real-time PCR.

We evaluated the following genes: paired box (*Pax*) 7 (#4331182, Hs 00242962_m1); myogenic differentiation (*MyoD*) 1 (#4331182, Hs 00159528_m1), *myogenin* (#4331182, Hs 01072232 GEX per-design_m1), and hypoxia-inducible factor (*HIF*; #4331182, Hs00153153_m). Glyceraldehyde-3-phosphate dehydrogenase (*GAPDH*; #4331182, Hs99999905_m1) was used as the internal control.

An Applied Biosystems Prism 7900HT Sequence Detection System was used, with the Sequence Detector Software (SDS version 2.0; Applied Biosystems). The relative quantification of the target gene derive from the 2^−ΔΔCt^ method.

### miRNA expression (miR-1, miR-133, miR-206)

PureLink miRNA Isolation kits were used for the miRNA extractions (Cat. No. K1570-01, Invitrogen, Life Technologies, Molecular Devices, Sunnyvale, USA). About 800,000 cells were resuspended in 300 μl binding buffer (the buffers were present in the PureLink miRNA kit), and 300 μl 70% alcohol was added to the lysate. This was forced into the spin cartridges of PureLink miRNA Isolation kits, which were then centrifuged at 12,000 × g for 1 min; after washing with 100% alcohol, these were centrifuged again, as before. Then 500 μl wash buffer was added to the spin cartridges, which were centrifuged again at 12,000 × g for 1 min. This procedure was performed twice, and then the spin cartridges were centrifuged at 12,000 × g for 3 min, to remove residual buffer. Finally, they were eluted with 50 μL RNase-free sterile water. The RNA concentrations were determined using a NanoDrop™ spectrophotometer. Retro-transcription and real-time PCR were carried out according to the Applied Biosystems TaqMan miRNA assay kit protocols. Briefly, the retro-transcription involved 20 ng of a “small” RNA, as the “stem loop” primer specific for each miRNA, dNTPs and inverse transcriptase RNAse inhibitors (according to the Applied Biosystems High capacity cDNA reverse transcription kit, part N° 4368814), using a Thermocycler (30 min at 16°C, 30 min at 42°C, 5 min at 85°C, then at 4°C). Then, real-time PCR for the miRNA expression levels was performed using the TaqMan probes and the specific TaqMan® Universal Master Mix II, no UNG, in 96-well plates (Part No.: 4440040, Applied Biosystems) with an Applied Biosystems PRISM 7900 HT Sequence Detection System, in triplicate. MiR-16 was used as the endogenous control. The specific miRNA sequence probes used (Applied Biosystems) were:

- has-miR-1 (UGGAAUGUAAAGAAGUAUGUAU; #002222);
- has-miR-206 (UGGAAUGUAAGGAAGUGUGUGG; #000510);
- has-miR-133b (UUUGGUCCCCUUCAACCAGCUA; #002247);
- has-miR-16-5p (UAGCAGCACGUAAAUAUUGGCG; #000391).

The relative quantification of the miRNA targets was carried out using the ΔCt formula, according to the Ct method.

## Statistical analysis

The statistical analysis was carried out using GraphPad Prism Software, version 5 (GraphPad Software, La Jolla, CA, USA). Some of the data are reported as means ± standard deviation or standard error, as mentioned where relevant. Unpaired *t*-tests were used to reveal statistical differences. The statistical significances indicated are ^*^p ≤ 0.05, ^**^p ≤ 0.005, and ^***^p ≤ 0.0001.

## Results

### Myogenic characteristics of female human myoblasts

The myoblasts were obtained from all of the muscle samples, as PRE-Hypoxia and POST-Hypoxia samples. The desmin-positive myoblasts decreased in all POST-Hypoxia samples compared to PRE-Hypoxia ones except for subject #3 (Table 1).

**Table 1:** **The myogenicity of satellite cell population**.

**Subject**	**Percentage myogenicity**	
	**PRE-hypoxia**	**POST-hypoxia**
#1	68	3
#2	66	33
#3	76	84
#4	90	88
#5	70	47
#6	67	8
#7	50	17
m ± SE	70 ± 4.5	40 ± 13^*^

*The Table reports the percentage of Desmin positive cells, as marker of myogenicity, in both conditions, PRE- and POST-Hypoxia samples. m ± SE, mean and standard error*.

* *POST-hypoxia vs. PRE-hypoxia, p < 0.02 (one tailed t-test)*.

### Immunohistochemistry on single fiber

**Figure 1:**
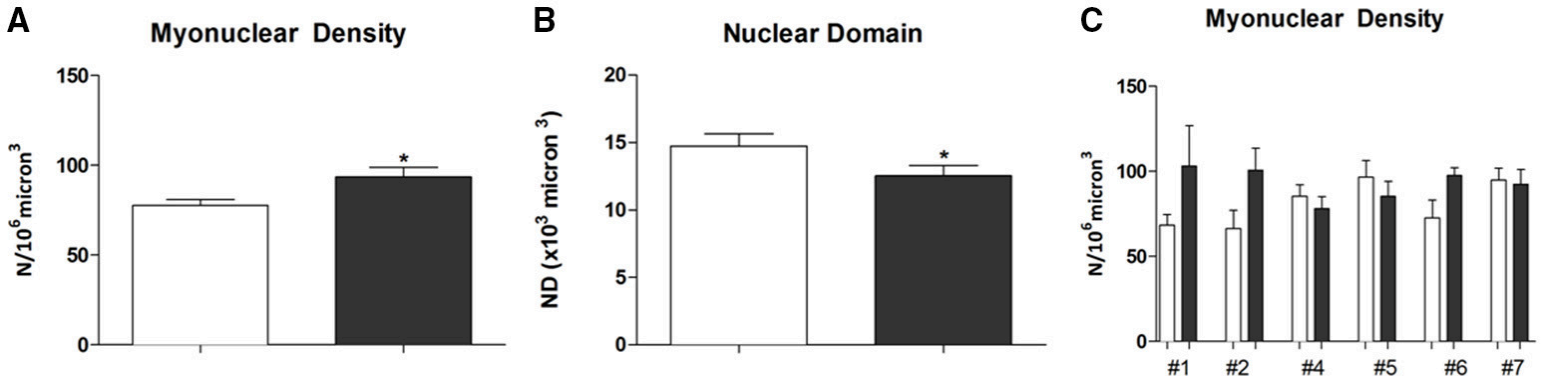
**Analysis of myonuclei on single fibers**. The number of myonuclei (N) in 10^6^ μm^3^ fiber volume, **(A)**, increased on POST-Hypoxia samples. The Nuclear Domain (ND), the fiber volume 10^3^ micron^3^ containing the nuclei, **(B)**, decreased at POST-Hypoxia samples. In **(C)** has been reported the myonuclear density counted in single fiber of each subject. The data are expressed as means and standard errors. Empty bars represent PRE-Hypoxia while dark bars POST-Hypoxia samples. ^*^p ≤ 0.05.

The myonuclei number measured on a constant volume (10^6^ μm^3^) were 77.7 ± 3.3 on PRE-Hypoxia (*n* = 56) and 93.5 ± 5.4 POST-Hypoxia (*n* = 59) fibers (*p* = 0.049; Figure 1A). The nuclear domain was decreased in POST-Hypoxia with respect to PRE-Hypoxia, 12.5 ± 0.7 × 10^3^ and 14.7 ± 0.9 × 10^3^ μm^3^, respectively (*p* = 0.048; Figure 1B). The myonuclear density analysis for single subject showed an increased number of nuclei for #1, #2, and #6 POST-Hypoxia fibers, whereas maintained similar number for #4, #5, and #7 POST-Hypoxia fibers with respect to PRE-Hypoxia ones (Figure 1C).

**Figure 2:**
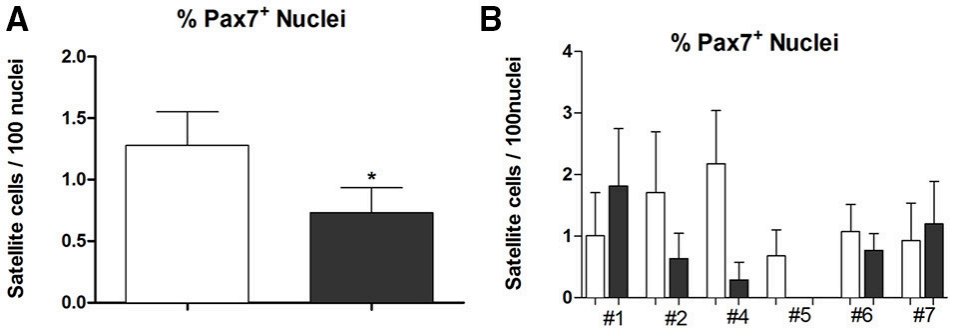
**The graph in (A) shows the percentage of Pax7^+^ nuclei as mean and standard error while in (B) the percentage of Pax7^+^ nuclei on single dissected fibers in each subject**. Empty bars represent PRE-Hypoxia while dark bars POST-Hypoxia samples. ^*^p ≤ 0.05.

Moreover, we found 1.27 ± 0.27 percentage of Pax7^+^ nuclei (*n* = 29) on PRE-Hypoxia and 0.75 ± 0.2 (*n* = 32) on POST-Hypoxia fibers (*p* = 0.045), a depletion on Pax7^+^ nuclei number on satellite cells (Figure 2).

The number of nuclei referred to single subject showed a little variation in #1, #6, and #7 samples, and a remarkable reduction (90–100% less than control) of Pax7^+^ cells in #2, #4, and #5 POST-Hypoxia samples.

### Superoxide anion and ROS production, SOD, and catalase antioxidant enzymatic activity

We measured the myoblast production of the ${\text{O}}_{2}^{•-}$ (Table 2): subjects #1 and #6 did not show ${\text{O}}_{2}^{•-}$ production alteration; subjects #3 and #4 had decreased the ${\text{O}}_{2}^{•-}$ production, while subjects #2 and #5 had increased ${\text{O}}_{2}^{•-}$ production comparing POST- vs. PRE-Hypoxia. The myoblast populations isolated from subjects #2 and #5 are named oxidized myoblats through the text.

**Table 2:** **The superoxide anion production**.

**Subject**	**Superoxide anion production**	
	**PRE-hypoxia**	**POST-hypoxia**
#1	0.17 ± 0.030	0.18 ± 0.010
#2	0.13 ± 0.012	0.21 ± 0.008^*^
#3	0.14 ± 0.004	0.10 ± 0.002^§^
#4	0.08 ± 0.004	0.05 ± 0.001^§^
#5	0.06 ± 0.002	0.20 ± 0.006^*^
#6	0.10 ± 0.003	0.09 ± 0.003

*The superoxide anion detection on the control (PRE-hypoxia) and hypobaric hypoxia exposed (POST-hypoxia) satellite cells, as revealed by the NBT dye fluorescence. Data are means ± standard deviation*.

* *Increased ${\text{O}}_{2}^{•-}$ radical level on POST-hypoxia vs. PRE-hypoxia, p < 0.0001*.

§ *Decreased ${\text{O}}_{2}^{•-}$ radical level on POST-hypoxia vs. PRE-hypoxia, p < 0.0001*.

**Figure 3:**
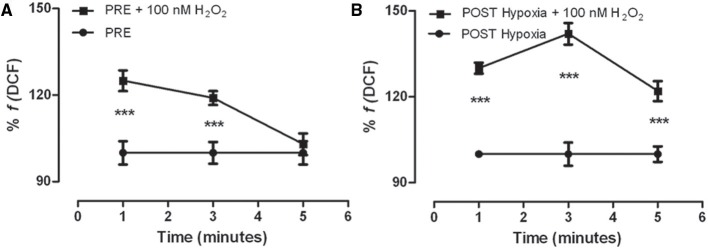
**Kinetics of DCF fluorescence in the control (PRE-Hypoxia; A) and POST-Hypoxia (B) satellite cells obtained from skeletal muscle of female subjects**. Both PRE- and POST-Hypoxia myoblasts were stimulated with 100 nM H_2_O_2_ (as indicated). The fluorescence of PRE- and POST-Hypoxia satellite cells at 0 min, that is without stimulation (point not shown), was considered as 100%. The fluorescence of PRE- and POST-Hypoxia cell control, without stimulation, remained stable during the time recording, and still reported as 100%. After stimulation, the cells showed a significant transient fluorescence increase reverted in 5 min only by PRE-Hypoxia cells. ^***^p ≤ 0.0001 vs. no H_2_O_2_-treated cells.

We investigated the H_2_O_2_ detoxification ability in #2 and #5 cell populations. The PRE-Hypoxia myoblasts were able to detoxify the added H_2_O_2_ in 5 min (Figure 3A), the POST-Hypoxia myoblasts were no (Figure 3B). The activity of antioxidant enzyme superoxide dismutase and catalase on oxidized myoblasts did not significantly change comparing POST- vs. PRE-Hypoxia (SOD: 27.9 ± 0.4 and 27.2 ± 0.5 U/ng protein; Cat: 7.7 ± 5.9 and 2.9 ± 2.2 U/ng protein; data not shown).

### Lipid peroxidation assay

**Figure 4:**
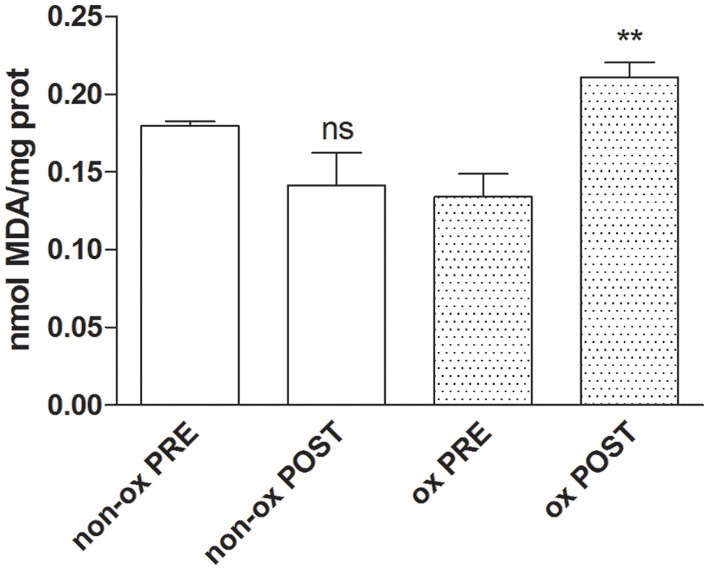
**The graph shows the increase of malondialdehyde values as maker of the lipid damage in oxidized POST-Hypoxia cells (dotted bars, ox POST)**. ^**^p ≤ 0.01.

We investigated the oxidative damage measuring the malondialdehyde concentration as marker of lipid peroxidation. The analysis revealed a significant increase of lipid damage on oxidized POST-Hypoxia cells with respect of both oxidized PRE-Hypoxia cells and non-oxidized PRE- and POST-Hypoxia cells (Figure 4).

### Analysis of differentiation

The differentiation of cells showed a significant decreased percentage of the fusion index comparing PRE–vs. POST–Hypoxia (46 ± 15 vs. 19 ± 12, *p* = 0.004). This result was particularly evident in oxidized myoblasts (#2: 60 PRE vs. 8 POST and #5: 45 PRE vs. 14% POST, data not shown).

### Analysis of transmembrane mitochondrial potential

**Figure 5:**
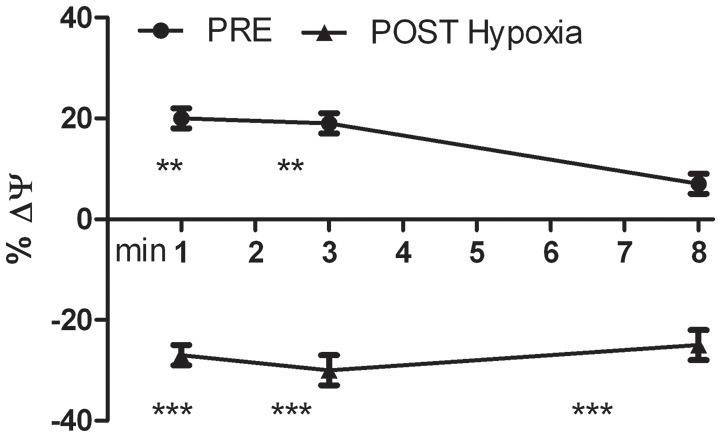
**Kinetics of the percentage variation of transmembrane mitochondrial potential (ΔΨ) of PRE- and POST-Hypoxia differentiated cells after stimulation with 100 nM H_2_O_2_**. The fluorescence of control cells for both PRE- and POST-Hypoxia conditions, were fixed as 0% and not showed in the graph. The PRE-Hypoxia cells after H_2_O_2_ stimulation showed a significant hyperpolarization of the transmembrane mitochondrial potentials while the POST-Hypoxia cells a significant depolarization. ^**^p ≤ 0.01, ^***^p ≤ 0.0001 vs. no H_2_O_2_-treated cells.

We analyzed the transmembrane mitochondrial potential of the living differentiated oxidized myoblasts. The JC-1 time course analysis showed that in PRE-Hypoxia cells after H_2_O_2_ stimulation the transmembrane mitochondrial potentials was initially hyperpolarized and at the experimental end point almost recovered the basal level. In contrast, the POST-Hypoxia cells were significantly and stably depolarized (Figure 5).

### Quantitative gene expression

We tested the gene expression of *Pax7, Myo, myogenin*, and *HIF* in undifferentiated and differentiated cells, except for *Myogenin* (data not shown).

*Pax7* gene, tested in undifferentiated cells, was highly up-regulated in non-oxidized cells (3.11 log_10_ RQ) while it resulted slightly up-regulated in the oxidized ones (0.17 log_10_ RQ).

In undifferentiated non-oxidized cells *MyoD* gene expression was up-regulated (3.2 log_10_ RQ) while it was down-regulated (−0.42 log_10_ RQ) in oxidized ones. Moreover, *MyoD* expression also decreased comparing non-oxidized undifferentiated vs. differentiated conditions (3.2 vs. 0.4 log_10_ RQ), while it increased comparing undifferentiated vs. differentiated in oxidized conditions (−0.42 vs. 3.11 log_10_ RQ).

*Myogenin* gene, which analysis was restricted to differentiated cells, was highly up-regulated in non-oxidized cells (3.45 log_10_ RQ) while it was down-regulated in the oxidized ones (−0.35 log_10_ RQ).

*HIF* expression was down-regulated in non-oxidized cells, both in undifferentiated and differentiated conditions (−0.007 and −0.38 log_10_ RQ, respectively); the same in oxidized undifferentiated cells (−0.03 log_10_ RQ) while in the differentiated the *HIF* was up-regulated (3.14 log_10_ RQ).

### Epigenetic profile induced by hypobaric hypoxic conditions

We analyzed the most important myo-miRNAs in terms of their regulation in the muscle cells: miR-1, miR-133b, and miR-206, while using miR-16 as the internal control. These myo-miRNAs are involved in proliferation/apoptosis (miR-1, miR-133b) and in differentiation (miR-206) of myogenic cells. We thus determined the expression of these miRNAs in the myoblasts during proliferation and differentiation.

**Figure 6:**
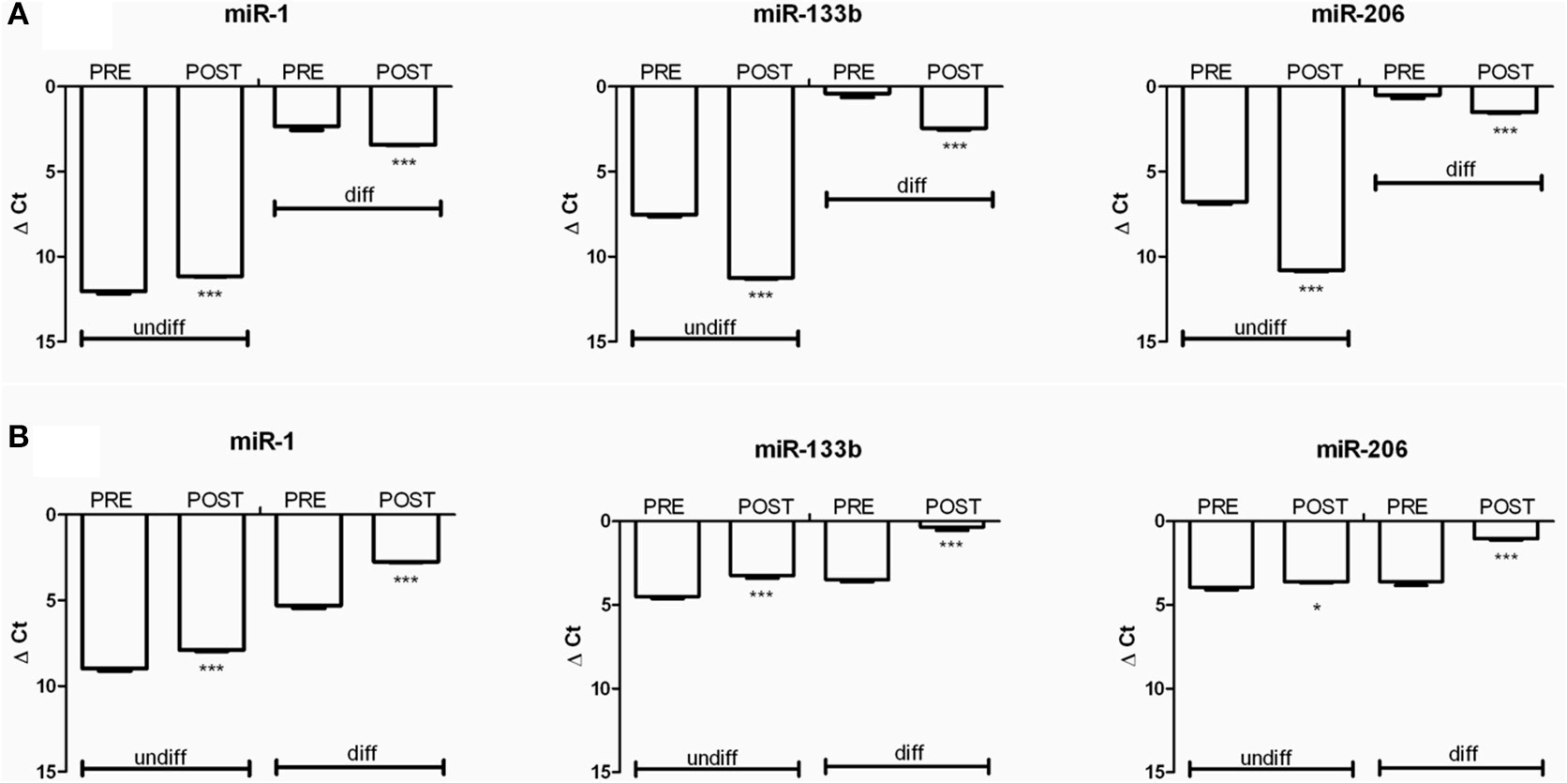
**The graphs show the relative expression of miR-1, miR-133b, and miR-206 before (PRE) and after (POST) Hymalaia expedition in cells with increased (A) and reduced (B) superoxide production**. The analysis were performed on undifferentiated and differentiated cells. **(A)** In oxidized cells, both in undifferentiated and differentiated conditions, the cells showed a down-regulation of miRNA 133b and miRNA 206. miRNA-1 was down-regulated in POST-Hypoxia differentiated cells while up-regulated in undifferentiated cells, comparing to PRE-Hypoxia. **(B)** In non-oxidized cells both in undifferentiated and differentiated conditions POST-Hypoxia cells showed an up-regulation of all tested miRNAs compared to PRE-Hypoxia cells. The independent experiments were performed in triplicates. ^*^p < 0.05 and ^***^p < 0.0001.

The Figure 6A, shows the miRNA regulation on oxidized POST-Hypoxia cells demonstrating a significant down-regulated expression of miR-133b in both undifferentiated and differentiated cells, with respect to the PRE-Hypoxia controls. The expression of miR-1 was significantly up-regulated in the undifferentiated POST-Hypoxia cells, while it was down-regulated in the differentiated POST-Hypoxia cells. The miR-206 expression in the undifferentiated and differentiated POST-Hypoxia cells were significantly down-regulated with respect to the control.

The Figure 6B shows the miRNA regulation on non-oxidized cell populations. We can observe that miR-1, miR-133b, and miR-206 were up-regulated in both undifferentiated and differentiated phenotype comparing POST- vs. PRE-Hypoxia.

## Discussion

To the best of our knowledge, there have been few studies conducted on skeletal muscle regeneration of women conditioned by a hypobaric hypoxic environment. We previously reported interesting data on skeletal muscle and satellite cell adaptation to hypobaric hypoxia for male climbers (Mancinelli et al., 2011). However, no similar studies have been published on sedentary females exposed to hypobaric hypoxia conditions, such as during the Himalayan Gokio Kumbu/Amadablam 2012 expedition. Evidence of physiological adaptation to high altitude has largely been investigated on men despite female subjects even if a sex as well as an individual dependent-response variability to hypobaric hypoxia seems to exist (Chapman et al., 1998; Mariggiò et al., 2010; Morabito et al., 2015; Tam et al., 2016).

In our previous study (Mancinelli et al., 2011), we demonstrated that after exercise under hypobaric hypoxia conditions, the yield of satellite cells from needle biopsies was nullified, except for the youngest climber. Specifically, the number of cells fused with existing fibers were increased after the expedition than before. In the present work, the yields of satellite cells from needle biopsies of female subjects after hypobaric hypoxia exposure revealed a significant decrease and also in this case the number of nuclei fused with existing fibers significantly increased. In fact, the number of Pax7^+^ nuclei, typical of activated satellite cells, decreased on POST-Hypoxia fibers. The increased myonuclear density and the decreased nuclear domain in POST-Hypoxia fibers indicate that satellite cells fusion into mature fibers occurred. Interestingly the oxidized POST-Hypoxia samples showed the most significant reduction of Pax7^+^ nuclei. This result is in accordance with data on male climbers, confirming that satellite cells support the demand of mature fiber for new nuclei in hypobaric-hypoxic condition.

These results might derive from the different genders, whereby hormonal condition of childbearing females could have influenced it (Fanò et al., 2001). Indeed, we analyzed the hormonal profile of female volunteers observing that they were all in the follicular phase of the menstrual cycle except one (non-oxidized sample) who was in the luteal phase (personal communication). So they were basically under the same hormonal asset and in our opinion this means that oxidation could not depend on it.

Hypobaric hypoxia exposure affects skeletal muscle metabolism from several points of view, and in particular, mitochondrial functions can be severely impaired (Magalhaes et al., 2005; Li et al., 2007).

The electron transfer for complete reduction of oxygen by mitochondrial complexes I and III-IV could be the main source of ROS, and specifically ${\text{O}}_{2}^{•-}$ (Muller et al., 2004). We have demonstrated that following the hypobaric hypoxia exposure the POST-Hypoxia samples responded differently in terms of the ${\text{O}}_{2}^{•-}$ production, surprisingly two out of six subjects showed an increase of 60 and 300% (oxidized cells) while the other did not (non-oxidized cells). We think that the exercise performed during the ascending could have had a beneficial role in managing oxidation, but further evidences need to be collected in favor of this hypothesis.

It is worth mentioning that trekking performed by the same subjects and at a similar intensity but at low altitude, did not produce so huge amount of ${\text{O}}_{2}^{•-}$ (Pietrangelo et al., 2015). Normally, the mitochondrial and cytoplasmatic superoxide dismutase activity converts the radical superoxide to H_2_O_2_, and then it is reduced by catalase (Jackson et al., 2007); however, we demonstrated that in the oxidized POST-Hypoxia myoblasts, both the superoxide dismutase and catalase activities did not vary and as a consequence a ${\text{O}}_{2}^{•-}$ and H_2_O_2_ could be accumulated within the cells that undergo oxidation. In fact, when we stimulated oxidized POST-Hypoxia cells using H_2_O_2_, they were not able to reduce it in 5 min. Moreover, the ${\text{O}}_{2}^{•-}$ accumulation provoked a significant lipid peroxidation in oxidized POST-Hypoxia cells.

The other important source of ${\text{O}}_{2}^{•-}$ production is the NADH oxidase activity that is linked to cytosolic NAD(H) redox (Mohazzab-H et al., 1997). Even if we cannot exclude a contribution from this source, based on our present data, we believe that the mitochondria have the main role in the oxidation of these myoblasts. It has been proposed that mitochondrial uncoupling represents a source for mitochondrial production of ROS (Negre-Salvayre et al., 1997; Sibille et al., 2001). Moreover, hypoxia induces the respiratory chain components into the maximally reduced redox state, due to a lower rate of reduction of oxygen at complex III (Abele et al., 2002). In this condition the mitochondrial uncoupling could be linked to marked decrease in the mitochondrial transmembrane electrical potential with alterations to the ATP/ADP ratio that in turn could further increase oxidation of the cell. Indeed, mitochondria are both the source and the target of the oxidation status. The analysis of mitochondrial transmembrane electrical potential in oxidized living myotubes revealed that POST-Hypoxia cells were significantly depolarized by acute H_2_O_2_ administration.

However, albeit we do not know the mechanism by which hypobaric hypoxia determines this mitochondria uncoupling and/or damage in cells, small depression in state III mitochondrial respiration and significant mitochondrial leakage on fibers of the same women has been demonstrated (Tam et al., 2016).

Here we investigated the effect of hypoxia on the ability of human myogenic cells to differentiate in culture. The inhibition of mouse myogenic differentiation under hypoxic conditions was already reported in literature. Di Carlo et al. (2004) demonstrated that hypoxia inhibited myogenic differentiation through acceleration of MyoD degradation by the ubiquitin-proteasome pathway that in turn blocked accumulation of Myogenin.

Furthermore, we demonstrated that exposure to hypoxia strongly inhibited also human myotube formation. We analyzed the expression of differentiation markers in our samples distinguishing between oxidized and non-oxidized cell populations. We found a specific effect linked to ${\text{O}}_{2}^{•-}$ accumulation. In fact, our results in MRF gene expression were in accordance with the normal myogenic process in non-oxidized POST-Hypoxia samples, while they were dysregulated in oxidized POST-Hypoxia samples.

We also analyzed the *HIF* expression, a known regulator factor that under normal oxygen tension is subject to oxygen-dependent ubiquitinylation and degradation by the 26S proteasome. On the other hand, under low oxygen tension, HIF-1α escapes degradation, and associates with nuclear HIF and, upon binding to specific coactivators, initiates transcription of HIF-responsive genes (Chandel et al., 2000). We supposed to find HIF expression not dysregulated in all our POST-Hypoxia samples do to the fact that we cultured them *in vitro* under normal oxygen tension. Unexpectedly, we found HIF up-regulated in oxidized POST-Hypoxia myotubes suggesting that the increase of ${\text{O}}_{2}^{•-}$ could affect the *HIF* expression. There is evidence that ROS and cytosolic H_2_O_2_ levels are involved in the stabilization/activation of HIF-1α (Chandel et al., 2000).

In our previous study, we have emphasized the role of miRNAs as novel post-transcriptional superoxide anion-sensible modulators (Pietrangelo et al., 2015). In fact, the increased ${\text{O}}_{2}^{•-}$ production down-regulated miR-133b and miR-206 expression both in differentiated and undifferentiated POST-hypoxia cells, in accordance with the miRNA results on myoblasts of the same subjects after trekking at low altitude, except for miR-1 that was up-regulated in present oxidized POST-hypoxia myoblasts. The specific miR-1 and miR-133b regulation could suggest the activation of apoptosis in the POST-Hypoxia oxidized myoblasts (Xu et al., 2007). Moreover, we think that this specific miR-1 and miR-133b expression is linked to ${\text{O}}_{2}^{•-}$ accumulation independently from the condition that trigger it. Indeed, similar epigenetic signature is in accordance with data collected both in human and canine myoblasts for which the oxidative stress was due to aging process (Sciancalepore et al., 2012; Fulle et al., 2013; La Rovere et al., 2014).

The miRNA expression in non-oxidized cells confirmed our hypothesis. In fact, all tested miRNA were found up-regulated both in undifferentiated and differentiated POST-hypoxia vs. PRE-Hypoxia cells as already found in the cells of the same subjects after similar trekking performed at low altitude (Pietrangelo et al., 2015).

The expression of miR-206 was in agreement with normal cell differentiation in all of the samples analyzed, which revealed that there was no modulation by the hypobaric hypoxia environment and by the superoxide production. However, the correct expression of miR-206 was not sufficient to promote correct POST-Hypoxia cell differentiation.

This is the first report that links female myogenic populations with hypobaric hypoxia-dependent and indipendent ${\text{O}}_{2}^{•-}$ production. In particular, our data suggest that it exists a personal adaptive capacity to oxidant balance. Of note, the understanding of the cellular adaptation to hypoxia-dependent oxidative stress, could be useful to highlight the dysregulated pathways found also in human diseases where etiopathology depend on hypoxia.

## Conclusions

In the present study, we have distinguished two adaptive phenomena for female human satellite cell populations under hypobaric hypoxia, responders, and non-responder female subjects have been identified in terms of ${\text{O}}_{2}^{•-}$ production and consequent oxidative stress. In particular, hypobaric-hypoxia increased the satellite cells fusion into mature fibers and impaired the differentiation process, with the oxidized cells resulted more affected. Moreover, the altered expression of muscle-specific transcription factors with specific expression of miR-1 and miR-133 revealed a specific genetic and epigenetic signature dependent on the oxidative status.

## Author contributions

RM designed the project, realized gene expression experiments, analyzed, and discussed the data, wrote the manuscript. ED performed experiments on oxidative status and miRNA regulation. VV organized and participated with the volunteers at the Hymalaian expedition and discussed the data. SF projected, analyzed, and discussed the data. LT and CR performed experiments on single fibers and discussed the data. TP designed the project, analyzed, and discussed the data, wrote the manuscript.

### Conflict of interest statement

The authors declare that the research was conducted in the absence of any commercial or financial relationships that could be construed as a potential conflict of interest.

## References

[B1] Abele D. Heise K. Portner H. O. Puntarulo S. (2002). Temperature dependence of mitochondrial function and production of reactive oxygen species in the intertidal mud clam. *Mya arenaria*. J. Exp. Biol. 205, 1831–1841. 12077159 10.1242/jeb.205.13.1831

[B2] Archer S. Michelakis E. (2002). The mechanism(s) of hypoxic pulmonary vasoconstriction: potassium channels, redox O_2_ sensors, and controversies. News Physiol. Sci. 17, 131–137. 10.1152/nips.01388.2002 12136039

[B3] Barbieri E. Sestili P. (2012). Reactive oxygen species in skeletal muscle signaling. J. Signal. Transduct. 2012:982794. 10.1155/2012/982794 22175016 PMC3235811

[B4] Barbieri E. Sestili P. Vallorani L. Guescini M. Calcabrini C. Gioacchini A. M.. (2014). Mitohormesis in muscle cells: a morphological, molecular, and proteomic approach. Muscles Ligaments Tendons J. 3, 254–266. 10.11138/mltj/2013.3.4.254 24596688 PMC3940498

[B5] Beccafico S. Puglielli C. Pietrangelo T. Bellomo R. Fanò G. Fulle S. (2007). Age-dependent effects on functional aspects in human satellite cells. Ann. N.Y. Acad. Sci. 1100, 345–352. 10.1196/annals.1395.037 17460197

[B6] Ceafalan L. C. Popescu B. O. Hinescu M. E. (2014). Cellular players in skeletal muscle regeneration. BioMed. Res. Int. 2014:957014. 10.1155/2014/957014 24779022 PMC3980925

[B7] Chandel N. S. McClintock D. S. Feliciano C. E. Wood T. M. Melendez J. A. Rodriguez A. M.. (2000). Reactive oxygen species generated at mitochondrial complex III stabilize hypoxia-inducible factor-1alpha during hypoxia: a mechanism of O_2_ sensing. J. Biol. Chem. 275, 25130–25138. 10.1074/jbc.M001914200 10833514

[B8] Chapman R. F. Stray-Gundersen S. Levine B. D. (1998). Individual variation in response to altitude training. J. Appl. Physiol. 85, 1448–1456. 9760340 10.1152/jappl.1998.85.4.1448

[B9] Di Carlo A. De Mori R. Martelli F. Pompilio G. Capogrossi M. C. Germani A. (2004). Hypoxia inhibits myogenic differentiation through accelerated MyoD degradation. J. Biol. Chem. 279, 16332–16338. 10.1074/jbc.M313931200 14754880

[B10] Di Filippo E. S. Mancinelli R. Pietrangelo T. La Rovere R. M. Quattrocelli M. Sampaolesi M.. (2016). Myomir dysregulation and reactive oxygen species in aged human satellite cells. Biochem. Biophys. Res. Commun. 473, 462–470. 10.1016/j.bbrc.2016.03.030 26975470

[B11] Doria C. Toniolo L. Verratti V. Cancellara P. Pietrangelo T. Marconi V.. (2011). Improved VO2 uptake kinetics and shift in muscle fiber type in high-altitude trekkers. J. Appl. Physiol. 111, 1597–1605. 10.1152/japplphysiol.01439.2010 21868681

[B12] Eisenberg I. Alexander M. S. Kunkel L. M. (2009). miRNAS in normal and diseased skeletal muscle. J. Cell. Mol. Med. 13, 2–11. 10.1111/j.1582-4934.2008.00524.x 19175696 PMC3072056

[B13] Fanò G. Mecocci P. Vecchiet J. Belia S. Fulle S. Polidori M. C.. (2001). Age and sex influence on oxidative damage and functional status in human skeletal muscle. J. Muscle Res. Cell. Motil. 22, 345–351. 10.1023/A:1013122805060 11808774

[B14] Fulle S. Di Donna S. Puglielli C. Pietrangelo T. Beccafico S. Bellomo R.. (2005). Age-dependent imbalance of the antioxidative system in human satellite cells. Exp. Gerontol. 40, 189–197. 10.1016/j.exger.2004.11.006 15763396

[B15] Fulle S. Mecocci P. Fanó G. Vecchiet I. Vecchini A. Racciotti D.. (2000). Specific oxidative alterations in vastus lateralis muscle of patients with the diagnosis of chronic fatigue syndrome. Free Radic. Biol. Med. 29, 1252–1259. 10.1016/S0891-5849(00)00419-6 11118815

[B16] Fulle S. Sancilio S. Mancinelli R. Gatta V. Di Pietro R. (2013). Dual role of the caspase enzymes in satellite cells from aged and young subjects. Cell Death Dis. 4:e955. 10.1038/cddis.2013.472 24336075 PMC3877545

[B17] Greenwald R. A. (1985). Therapeutic benefits of oxygen radical scavenger treatments remain unproven. J. Free Radic. Biol. Med. 1, 173–177. 10.1016/0748-5514(85)90115-1 3915304

[B18] Hoppeler H. Vogt M. Weibel E. R. Flück M. (2003). Response of skeletal muscle mitochondria to hypoxia. Exp. Physiol. 88, 109–119. 12525860 10.1113/eph8802513

[B19] Huang Z.-P. Espinoza-Lewis R. Wang D.-Z. (2012). Determination of miRNA targets in skeletal muscle cells. Methods Mol. Biol. 798, 475–490. 10.1007/978-1-61779-343-1_28 22130855 PMC4275444

[B20] Jackson M. J. Pye D. Palomero J. (2007). The production of reactive oxygen and nitrogen species by skeletal muscle. J. Appl. Physiol. 102, 1664–1670. 10.1152/japplphysiol.01102.2006 17082364

[B21] Kietzmann T. Fandrey J. Acker H. (2000). Oxygen radicals as messengers in oxygen-dependent gene expression. News Physiol. Sci. 15, 202–208. 11390911 10.1152/physiologyonline.2000.15.4.202

[B22] Kirkman H. N. Gaetani G. F. (2007). Mammalian catalase: a venerable enzyme with new mysteries. Trends Biochem. Sci. 32, 44–50. 10.1016/j.tibs.2006.11.003 17158050

[B23] La Rovere R. M. Quattrocelli M. Pietrangelo T. Di Filippo E. S. Maccatrozzo L. Mascarello F.. (2014). Myogenic potential of canine craniofacial satellite cells. Front. Aging Neurosci. 6:90. 10.3389/fnagi.2014.00090 24860499 PMC4026742

[B24] Li X. Zhu L. Chen X. Fan M. (2007). Effects of hypoxia on proliferation and differentiation of myoblasts. Med. Hypotheses 69, 629–636. 10.1016/j.mehy.2006.12.050 17395396

[B25] Magalhaes J. Ascensão A. Soares M. C. Ferreira R. Neuparth M. J. Marques F.. (2005). Acute and severe hypobaric hypoxia increases oxidative stress and impairs mitochondrial function in mouse skeletal muscle. J. Appl. Physiol. 99, 1247–1253. 10.1152/japplphysiol.01324.2004 15905323

[B26] Magenta A. Greco S. Gaetano C. Martelli F. (2013). Oxidative stress and microRNAs in vascular diseases. Int. J. Mol. Sci. 14, 17319–17346. 10.3390/ijms140917319 23975169 PMC3794730

[B27] Mancinelli R. Pietrangelo T. La Rovere R. Toniolo L. Fanò G. Reggiani C.. (2011). Cellular and molecular responses of human skeletal muscle exposed to hypoxic environment. J. Biol. Regul. Homeost. Agents 25, 635–645. 22217995

[B28] Mariggiò M. A. Falone S. Morabito C. Guarnieri S. Mirabilio A. Pilla R. (2010). Peripheral blood lymphocytes: a model for monitoring physiological adaptation to high altitude. High Alt. Med. Biol. 10, 333–342. 10.1089/ham.2009.1097 21190502

[B29] Menghini L. Leporini L. Scanu N. Pintore G. La Rovere R. Di Filippo E. S.. (2011). Effect of phytochemical concentrations on biological activities of cranberry extracts. J. Biol. Regul. Homeost. Agents 25, 27–35. 21382271

[B30] Mohazzab-H K. M. Kaminski P. M. Wolin M. S. (1997). Lactate and PO_2_ modulate superoxide anion production in bovine cardiac myocytes: potential role of NADH oxidase. Circulation 96, 614–620. 9244234 10.1161/01.cir.96.2.614

[B31] Morabito C. Lanuti P. Caprara G. A. Guarnieri S. Verratti V. Ricci G.. (2015). Responses of peripheral blood mononuclear cells to moderate exercise and hypoxia. Scand. J. Med. Sci. Sports. 10.1111/sms.12557. [Epub ahead of print]. 26432186

[B32] Morabito C. Rovetta F. Bizzarri M. Mazzoleni G. Fanò G. Mariggiò M. A. (2010). Modulation of redox status and calcium handling by extremely low frequency electromagnetic fields in C2C12 muscle cells: a real-time, single-cell approach. Free Radic Biol. Med. 48, 579–589. 10.1016/j.freeradbiomed.2009.12.005 20005945

[B33] Muller F. L. Liu Y. Van Remmen H. (2004). Complex III releases superoxide to both sides of inner mitochondrial membrane. J. Biol. Chem. 279, 49064–49073. 10.1074/jbc.M407715200 15317809

[B34] Negre-Salvayre A. Hirtz C. Carrera G. Cazenave R. Troly M. Salvayre R.. (1997). A role for uncoupling protein-2 as a regulator of mitochondrial hydrogen peroxide generation. FASEB J. 11, 809–815. 9271366

[B35] Nuydens R. Novalbos J. Dispersyn G. Weber C. Borgers M. Geerts H. (1999). A rapid method for the evaluation of compounds with mitochondria-protective properties. J. Neurosci. Methods 92, 153–159. 10595713 10.1016/s0165-0270(99)00107-7

[B36] Pietrangelo T. D'Amelio L. Doria C. Mancinelli R. Fulle S. Fanò G. (2011). Tiny percutaneous needle biopsy: an efficient method for studying cellular and molecular aspects of skeletal muscle in humans. Int. J. Mol. Med. 27, 361–367. 10.3892/ijmm.2010.582 21165550

[B37] Pietrangelo T. Di Filippo E. S. Mancinelli R. Doria C. Rotini A. Fanò-Illic G.. (2015). Low intensity exercise training improves skeletal muscle regeneration potential. Front. Physiol. 6:399. 10.3389/fphys.2015.00399 26733888 PMC4689811

[B38] Pietrangelo T. Perni S. Di Tano G. Fanò-Illic G. Franzini-Armstrong C. (2013). A method for the ultrastructural preservation of tiny percutaneous needle biopsy material from skeletal muscle. Int. J. Mol. Med. 32, 965–970. 10.3892/ijmm.2013.1454 23900509 PMC3812242

[B39] Pietrangelo T. Puglielli C. Mancinelli R. Beccafico S. Fanò G. Fulle S. (2009). Molecular basis of the myogenic profile of aged human skeletal muscle satellite cells during differentiation. Exp. Gerontol. 44, 523–531. 10.1016/j.exger.2009.05.002 19457451

[B40] Sciancalepore M. Luin E. Parato G. Ren E. Giniatullin R. Fabbretti E.. (2012). Reactive oxygen species contribute to the promotion of the ATP-mediated proliferation of mouse skeletal myoblasts. Free Radic. Biol. Med. 53, 1392–1398. 10.1016/j.freeradbiomed.2012.08.002 22917975

[B41] Sibille B. Filippi C. Piquet M. -A. Leclercq P. Fontaine E. Ronot X.. (2001). The mitochondrial consequences of uncoupling intact cells depend on the nature of the exogenous substrate. Biochem. J. 355, 231–235. 10.1042/bj3550231 11256968 PMC1221731

[B42] Tam E. Bruseghini P. Calabria E. Sacco L. D. Doria C. Grassi B.. (2016). Gokyo Khumbu/Ama Dablam Trek 2012: effects of physical training and high-altitude exposure on oxidative metabolism, muscle composition, and metabolic cost of walking in women. Eur. J. Appl. Physiol. 116, 129–144. 10.1007/s00421-015-3256-z 26349745

[B43] Vanden Hoek T. L. Becker L. B. Shao Z. Li C. Schumacker P. T. (1998). Reactive oxygen species released from mitochondria during brief hypoxia induce preconditioning in cardiomyocytes. J. Biol. Chem. 273, 18092–18098. 9660766 10.1074/jbc.273.29.18092

[B44] Verdijk L. B. (2014). Satellite cells activation as a critical step in skeletal muscle plasticity. Exp. Physiol. 99, 1449–1450. 10.1113/expphysiol.2014.081273 25362647

[B45] Verdijk L. B. Snijders T. Drost M. Delhaas T. Kadi F. van Loon L. J. (2014). Satellite cells in human skeletal muscle; from birth to old age. Age 36, 545–547. 10.1007/s11357-013-9583-2 24122288 PMC4039250

[B46] Verratti V. Falone S. Doria C. Pietrangelo T. Di Giulio C. (2015). Kilimanjaro Abruzzo expedition: effects of high-altitude trekking on anthropometric, cardiovascular and blood biochemical parameters. Sport Sci. Health 11, 271–278. 10.1007/s11332-015-0235-z 26613007 PMC4648976

[B47] Xu C. Lu Y. Pan Z. Chu W. Luo X. Lin H.. (2007). The muscle-specific microRNAs miR-1 and miR-133 produce opposing effects on apoptosis by targeting HSP60, HSP70 and caspase-9 in cardiomyocytes. J. Cell Sci. 120, 3045–3052. 10.1242/jcs.010728 17715156

